# Biomarker discovery using NUcleic Acid-Linked Immuno-Sandwich Assay in multiple sclerosis patients experiencing progression independent of relapse activity

**DOI:** 10.1177/13524585251375780

**Published:** 2025-10-14

**Authors:** Sofia Sandgren, Aleksandra Maleska Maceski, Pascal Benkert, Maximilian Einsiedler, Sabine Schaedelin, Johanna Oechtering, Lutz Achtnichts, Patrice H Lalive, Stefanie Müller, Caroline Pot, Amanda Heslegrave, David Hunt, Jan Lycke, Robert Hoepner, Patrick Roth, Claudio Gobbi, Manuel Comabella, Tobias Derfuss, Ludwig Kappos, Cristina Granziera, Ahmed Abdelhak, David Leppert, Eline AJ Willemse, Henrik Zetterberg, Jens Kuhle

**Affiliations:** Department of Clinical Neuroscience, Institute of Neuroscience and Physiology, Sahlgrenska Academy, University of Gothenburg, Gothenburg, Sweden; Department of Neurology, Sahlgrenska University Hospital, Region Västra Götaland, Gothenburg, Sweden; Multiple Sclerosis Centre and Research Center for Clinical Neuroimmunology and Neuroscience Basel (RC2NB), Departments of Biomedicine and Clinical Research, University Hospital and University of Basel, Basel, Switzerland; Department of Neurology, University Hospital and University of Basel, Basel, Switzerland; Multiple Sclerosis Centre and Research Center for Clinical Neuroimmunology and Neuroscience Basel (RC2NB), Departments of Biomedicine and Clinical Research, University Hospital and University of Basel, Basel, Switzerland; Department of Neurology, University Hospital and University of Basel, Basel, Switzerland; Multiple Sclerosis Centre and Research Center for Clinical Neuroimmunology and Neuroscience Basel (RC2NB), Departments of Biomedicine and Clinical Research, University Hospital and University of Basel, Basel, Switzerland; Department of Neurology, University Hospital and University of Basel, Basel, Switzerland; Multiple Sclerosis Centre and Research Center for Clinical Neuroimmunology and Neuroscience Basel (RC2NB), Departments of Biomedicine and Clinical Research, University Hospital and University of Basel, Basel, Switzerland; Department of Neurology, University Hospital and University of Basel, Basel, Switzerland; Multiple Sclerosis Centre and Research Center for Clinical Neuroimmunology and Neuroscience Basel (RC2NB), Departments of Biomedicine and Clinical Research, University Hospital and University of Basel, Basel, Switzerland; Department of Neurology, University Hospital and University of Basel, Basel, Switzerland; Department of Neurology, Cantonal Hospital Aarau, Aarau, Switzerland; Department of Clinical Neurosciences, Division of Neurology, and Department of Medicine, Translational Biomarker Group, Geneva, Switzerland; Division of Laboratory Medicine, Department of Diagnostic, Geneva University Hospitals, Geneva, Switzerland; Department of Pathology and Immunology, Faculty of Medicine, University of Geneva, Geneva, Switzerland; Department of Neurology, University Teaching and Research Hospital St. Gallen, St. Gallen, Switzerland; Service of Neurology and Laboratories of Neuroimmunology, Department of Clinical Neurosciences, Lausanne University Hospital (CHUV) and University of Lausanne, Lausanne, Switzerland; UK Dementia Research Institute at UCL, London, UK; Department of Neurodegenerative Disease, UCL Queen Square Institute of Neurology, London, UK; UK Dementia Research Institute, Centre for Clinical Brain Sciences, University of Edinburgh, Edinburgh, UK; Department of Clinical Neuroscience, Institute of Neuroscience and Physiology, Sahlgrenska Academy, University of Gothenburg, Gothenburg, Sweden; Department of Neurology, Sahlgrenska University Hospital, Region Västra Götaland, Gothenburg, Sweden; Department of Neurology, Inselspital, Bern University Hospital, University of Bern, Bern, Switzerland; Department of Neurology and Clinical Neuroscience Center, University Hospital Zurich and University of Zurich, Zurich, Switzerland; Multiple Sclerosis Center, Neurocenter of Southern Switzerland, EOC, Lugano, Switzerland; Faculty of Biomedical Sciences, Università della Svizzera Italiana (USI), Lugano, Switzerland; Servei de Neurologia, Centre d'Esclerosi Múltiple de Catalunya (Cemcat), Institut de Recerca Vall d'Hebron (VHIR), Hospital Universitari Vall d'Hebron, Universitat Autònoma de Barcelona, Barcelona, Spain; Multiple Sclerosis Centre and Research Center for Clinical Neuroimmunology and Neuroscience Basel (RC2NB), Departments of Biomedicine and Clinical Research, University Hospital and University of Basel, Basel, Switzerland; Department of Neurology, University Hospital and University of Basel, Basel, Switzerland; Multiple Sclerosis Centre and Research Center for Clinical Neuroimmunology and Neuroscience Basel (RC2NB), Departments of Biomedicine and Clinical Research, University Hospital and University of Basel, Basel, Switzerland; Department of Neurology, University Hospital and University of Basel, Basel, Switzerland; Multiple Sclerosis Centre and Research Center for Clinical Neuroimmunology and Neuroscience Basel (RC2NB), Departments of Biomedicine and Clinical Research, University Hospital and University of Basel, Basel, Switzerland; Department of Neurology, University Hospital Basel, University of Basel, Basel, Switzerland; Translational Imaging in Neurology (ThINK) Basel, Department of Biomedical Engineering, Faculty of Medicine, University Hospital Basel and University of Basel, Basel, Switzerland; Department of Neurology and Weill Institute for Neurosciences, University of California, San Francisco, San Francisco, CA, USA; Multiple Sclerosis Centre and Research Center for Clinical Neuroimmunology and Neuroscience Basel (RC2NB), Departments of Biomedicine and Clinical Research, University Hospital and University of Basel, Basel, Switzerland; Department of Neurology, University Hospital and University of Basel, Basel, Switzerland; Multiple Sclerosis Centre and Research Center for Clinical Neuroimmunology and Neuroscience Basel (RC2NB), Departments of Biomedicine and Clinical Research, University Hospital and University of Basel, Basel, Switzerland; Department of Neurology, University Hospital and University of Basel, Basel, Switzerland; UK Dementia Research Institute at UCL, London, UK; Department of Neurodegenerative Disease, UCL Queen Square Institute of Neurology, London, UK; Department of Psychiatry and Neurochemistry, Institute of Neuroscience and Physiology, The Sahlgrenska Academy at the University of Gothenburg, Mölndal, Sweden; Clinical Neurochemistry Laboratory, Sahlgrenska University Hospital, Mölndal, Sweden; Hong Kong Center for Neurodegenerative Diseases, Clear Water Bay, Hong Kong, China; Wisconsin Alzheimer’s Disease Research Center, University of Wisconsin School of Medicine and Public Health, University of Wisconsin-Madison, Madison, WI, USA; Multiple Sclerosis Centre and Research Center for Clinical Neuroimmunology and Neuroscience Basel (RC2NB), Departments of Biomedicine and Clinical Research, University Hospital and University of Basel, Basel, Switzerland; Department of Neurology, University Hospital and University of Basel, Basel, Switzerland

**Keywords:** Progression independent of relapse activity (PIRA), NUcleic Acid-Linked Immuno-Sandwich Assay (NULISA), multiple sclerosis (MS), blood-based biomarkers, glial fibrillary acidic protein (GFAP)

## Abstract

**Background/Objectives:**

This cohort study aimed to identify blood-based biomarkers associated with progression independent of relapse activity (PIRA) in persons with multiple sclerosis (pwMS) using the multiplex NUcleic Acid-Linked Immuno-Sandwich Assay (NULISA).

**Methods:**

NULISA ‘CNS Disease Panel’ and ‘Inflammation Panel’ were applied on plasma samples from pwMS following B cell-depleting therapy (BCDT; *n* = 185) or fingolimod (*n* = 200), median follow-up 4.0 (BCDT) and 9.1 (fingolimod) years. Plasma NULISA results (322 unique proteins; 0.9 and 1 year after treatment start, respectively) were investigated for their potential to prognosticate PIRA.

**Results:**

‘CNS Disease Panel’ derived glial fibrillary acidic protein (GFAP) and neurofilament light chain (NfL) were identified as predictive of PIRA by multivariable Cox regression models (GFAP: BCDT: hazard ratio (HR) = 1.79, 95% confidence interval (CI) = 1.26–2.55, *p* = 0.0011; fingolimod: HR = 1.73, 95% CI = 1.14–2.64, *p* = 0.0104; NfL: BCDT: HR = 1.99, 95% CI = 1.31–3.02, *p* = 0.0013). GFAP derived from the ‘Inflammation Panel’ exhibited patterns like those observed with ‘CNS Disease Panel’ derived GFAP. Beyond GFAP and NfL, 12 biomarker candidates predictive of PIRA were identified. No target passed multiple test corrections, but GFAP consistently showed the highest hazard.

**Conclusion:**

Among over 300 proteins investigated by NULISA, GFAP was the main biomarker significantly associated with future PIRA risk in our cohort.

## Introduction

Multiple sclerosis (MS) affects the central nervous system (CNS) and has two major pathophysiological hallmarks: neuroinflammation and neurodegeneration. Current treatments effectively suppress overt inflammatory disease activity in many persons with MS (pwMS), however they often experience progression independent of relapse activity (PIRA),^1^ which is a major contributor to cumulative disability in MS.^2^

The major objective of future therapeutic strategies is to prevent PIRA and the broader concept of smouldering-associated worsening (SAW).^3^ Understanding and quantifying SAW driven by neurodegeneration or other pathological processes that cause neural dysfunction is a significant unmet need in MS. The ability to capture the pathological processes in MS using body fluid biomarkers, for example, in an accessible compartment such as blood, may improve our understanding of the underlying mechanisms and the development of new therapeutic interventions.

We have identified and confirmed serum glial fibrillary acidic protein (sGFAP) as a promising biomarker of disease progression in MS^4–6^ and serum neurofilament light chain (sNfL) is also, albeit to a lesser degree associated with MS disease progression.^7,8^ However, more blood biomarkers are needed for individualized patient management and disease monitoring. With recent advancements in ultra-sensitive assays, such as NUcleic Acid-Linked Immuno-Sandwich Assay (NULISA) proteomics,^9^ advanced methods for protein analysis and biomarker detection have been developed, but not yet explored in deeply characterized MS cohorts with sufficiently long follow-up.

We aimed to confirm GFAP and NfL as biomarkers for predicting PIRA using the NULISA platform, and to explore new biomarkers beyond GFAP and NfL with the potential to predict PIRA. We used two well-characterized longitudinal pwMS cohorts, who were followed for nearly half a decade and a full decade, respectively, after the initiation of B cell-depleting therapy (BCDT) or fingolimod, with available Single molecule array (Simoa) derived concentrations for sGFAP and sNfL for comparative analysis.

## Methods

### Study design, study population and clinical endpoints

This study included pwMS who had started treatment with anti-CD20 monoclonal antibodies (BCDT cohort) or fingolimod (fingolimod cohort) from January 1, 2012, to October 22, 2022, in the Swiss MS Cohort (SMSC).^10,11^ Eligible pwMS were those with at least three clinical visits and follow-up at the University Hospital Basel (i.e. included cohorts constitute a subgroup of the previously described BCDT^5^ and fingolimod^6^ cohorts). For each patient an index (i.e. baseline) blood sample under stable treatment was selected 8–24 months (median 0.9 (BCDT) and 1.0 (fingolimod) years) after treatment start.

Clinical endpoint was time to PIRA. Data were extracted from the SMSC May 29, 2024. A description of the SMSC is available in Supplement. This study followed the STROBE reporting guidelines.

### Clinical measures: disability worsening and PIRA

Confirmed disability worsening (CDW) was defined as increase in the Expanded Disability Status Scale (EDSS)^12^ score by ⩾1.5, ⩾1, and ⩾0.5 if baseline EDSS was 0, 1.0–5.0, and ⩾5.5, respectively, confirmed at a subsequent visit ⩾6 months later. Progression independent of relapse activity (PIRA) was conservatively defined as CDW events without relapses between the reference and confirmation visit.^5^ For detailed definitions of CDW and PIRA, see the Supplement.

### Body fluid biomarkers

Blood samples were collected, processed onsite to isolate serum, or plasma, as appropriate, aliquoted, and stored at -80 °C.

### NUcleic acid Linked Immuno-Sandwich Assay analysis

The NULISA assays were conducted at Alamar Biosciences as previously outlined.^9^ A summary of the NULISA workflow is given in Supplement.

Fifty-two proteins were shared between the two panels, resulting in the examination of 322 unique proteins. Targets included in the ‘CNS Disease Panel’ (124 targets), the ‘Inflammation Panel’ (250 targets), and overlapping proteins are provided in Tables S1–S3 in the Supplement.

### Single molecule array (Simoa) analysis

Serum GFAP and NfL were measured using the Neurology 2-plex B assay (Quanterix, Billerica, MA) and Z scores adjusted for age, body mass index (BMI), and gender (GFAP only) based on a large healthy control cohort calculated as previously described.^5,6,13^

### Statistical analyses

Demographic and clinical characteristics were described as counts and percentages as well as median and interquartile range [IQR], as appropriate. pwMS who switched or discontinued treatment after initiation of disease-modifying therapy were retained in the risk set for future PIRA.

Target NULISA Protein Quantification (NPQ) units obtained in the NULISA analyses were used in individual (separately for each target) multivariable Cox regression models (adjusted for age, BMI, and gender) to assess their influence on time to PIRA. The resulting hazard ratios (HRs) represent the hazard of PIRA per 1-unit increase in log2(NPQ), corresponding to a doubling of the underlying normalized protein concentration. Both un-adjusted and adjusted for multiple comparison (i.e. false discovery rate (FDR)-adjusted^14^) p-values were reported.

The association between sGFAP and sNfL biomarker Z scores and time to a first PIRA event in pwMS (BCDT cohort and fingolimod cohort separately) was investigated using Kaplan-Meier curves and by multivariable Cox regression models (adjusted for age, BMI, and gender) using biomarker absolute values (to allow equal comparison between biomarkers since Z scores based on healthy controls^5,6,13^ are not available for NULISA analytes). In these Cox regression models, absolute biomarkers were log2-transformed and therefore the HRs reflect the hazard per doubling in biomarker concentration. In the BCDT cohort, a biomarker Z score cut-off of 1 was used (corresponding to 1 standard deviation above the mean of the reference population (= 84.1st percentile)), while a Z score cut-off of 0.75 was used for sGFAP in the fingolimod cohort to have a comparable number of low and high samples as for sNfL.^6^ Accordingly, sGFAP and sNfL Z scores >1 were categorized as ‘high’ and scores ⩽ 1 as ‘low’ in the BCDT cohort, whereas sGFAP Z scores > 0.75 were categorized as ‘high’ and ⩽ 0.75 as ‘low’, and sNfL Z scores > 1.0 as ‘high’ and ⩽ 1.0 as ‘low’ in the fingolimod cohort.

Correlations between log2-transformed sGFAP and sNfL levels (Simoa) and GFAP and NfL NPQ units (NULISA) were calculated using Pearson correlation coefficient (r). p-values ⩽ 0.05 were considered statistically significant. Analyses were performed in R version 4.3.1.

## Results

### Study population

#### BCDT cohort

The cohort consisted of 185 pwMS (70.3% RRMS; 17.3% SPMS; 12.4% PPMS) receiving ocrelizumab (67.6%) or rituximab (32.4%) with median [IQR] age of 43.9 [34.2, 53.1] years and EDSS 3.5 [2.0, 5.0] at baseline (Table 1). Index samples were obtained median 0.9 years after BCDT start, with median follow-up time 4.0 [2.6, 5.1] years after the index sample. During follow-up, 33% (*n* = 61/185) of pwMS experienced a PIRA event, 73.5% continued BCDT, 21.1 % discontinued or switched between BCDTs, whereas 5.4% switched to another type of MS drug (i.e. not a BCDT).

**Table 1.** Patient characteristics at start of BCDT and fingolimod treatment.

	BCDT (*n* = 185)	Fingolimod (*n* = 200)
Variable		
Gender = Women	118 (63.8%)	131 (65.5%)
Age [Y]	43.9 [34.2, 53.1]	41.4 [33.4, 48.1]
Disease subtype		
RRMS	130 (70.3%)	200 (100.0%)
SPMS	32 (17.3%)	0 (0.0%)
PPMS	23 (12.4%)	0 (0.0%)
Disease duration [Y]	10.7 [3.9, 19.9]	8.2 [2.7, 15.0]
BCDT/fingolimod start to index sample [Y]	0.9 [0.9, 1.0]	1.0 [0.9, 1.1]
BCDT drugs = ocrelizumab	125 (67.6%)	NA
Relapse < 4M before BCDT/fingolimod start	47 (25.4%)	65 (32.5%)
Relapse < 1Y before BCDT/fingolimod start	77 (41.6%)	108 (54.0%)
At index sample		
NfL [pg/ml]	8.9 [6.7, 13.4]	7.9 [5.7, 11.4]
NfL Z score	0.6 [-0.3, 1.4]	0.4 [-0.6, 1.3]
NfL Z score > 1 = TRUE	70 (37.8%)	69 (34.5%)
GFAP [pg/ml]	91.7 [64.9, 128.7]	79.5 [55.9, 104.2]
GFAP Z score	0.4 [-0.7, 1.4]	0.2 [-0.6, 1.0]
GFAP Z score: BCDT > 1; fingolimod > 0.75 = TRUE	68 (36.8%)	65 (32.5%)
Follow-up		
Still on BCDT/fingolimod at last follow-up	136 (73.5%)	95 (47.5%)
PIRA during follow-up	61 (33.0%)	69 (35.0%)

Reported values are *n* (%) and median [IQR] if not stated otherwise. BCDT, B cell-depleting treatment; EDSS, Expanded Disability Status Scale; FU, follow-up; GFAP, glial fibrillary acidic protein; IQR, interquartile range; M, months; NA, not applicable; NfL, neurofilament light chain; PIRA, progression independent of relapse activity; PPMS, primary progressive multiple sclerosis; RRMS, relapsing-remitting multiple sclerosis; SMSC, Swiss MS Cohort; SPMS, secondary progressive multiple sclerosis; Y, year.

#### Fingolimod cohort

The cohort consisted of 200 pwMS (all RRMS) receiving fingolimod with median [IQR] age of 41.4 [33.4, 48.1] years and an EDSS of 2.0 [1.5, 3.1] at baseline (Table 1). Index samples were obtained median 1.0 years after fingolimod start, with median follow-up time 9.1 [7.4, 10.6] years after the index sample. During follow-up, 35% (*n* = 69/200) of pwMS experienced a PIRA event, 47.5% continued fingolimod, 31% changed to monoclonal antibodies, 18.5% switched to platform or oral disease-modifying therapy, and 3.0% ended fingolimod without restarting.

### GFAP and NfL as biomarkers for predicting PIRA using NULISA (Simoa as reference)

#### BCDT cohort

**Figure 1.**
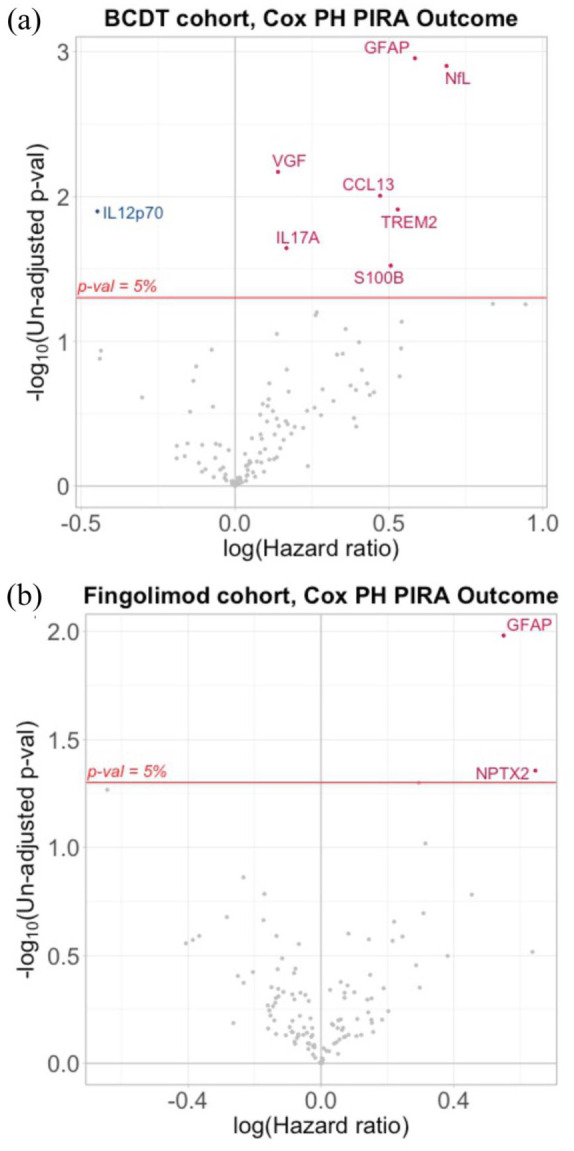
NULISA ‘CNS Disease Panel’. Volcano plot of all proteins tested in NULISA ‘CNS Disease Panel’ visualizing HR for PIRA prediction and statistical significance for (a) BCDT and (b) fingolimod cohort. Red dots indicate proteins predictive of subsequent PIRA, blue dots proteins protective of future PIRA, and grey dots proteins not reaching the un-adjusted p-value threshold of 0.05. The horizontal red line indicates the un-adjusted p-value threshold of 0.05. BCDT, B-cell depleting therapy; CNS, central nervous system; HR, hazard ratio; NULISA, NUcleic acid Linked Immuno-Sandwich Assay; PH, proportional-hazards; PIRA, progression independent of relapse activity.

**Figure 2.**
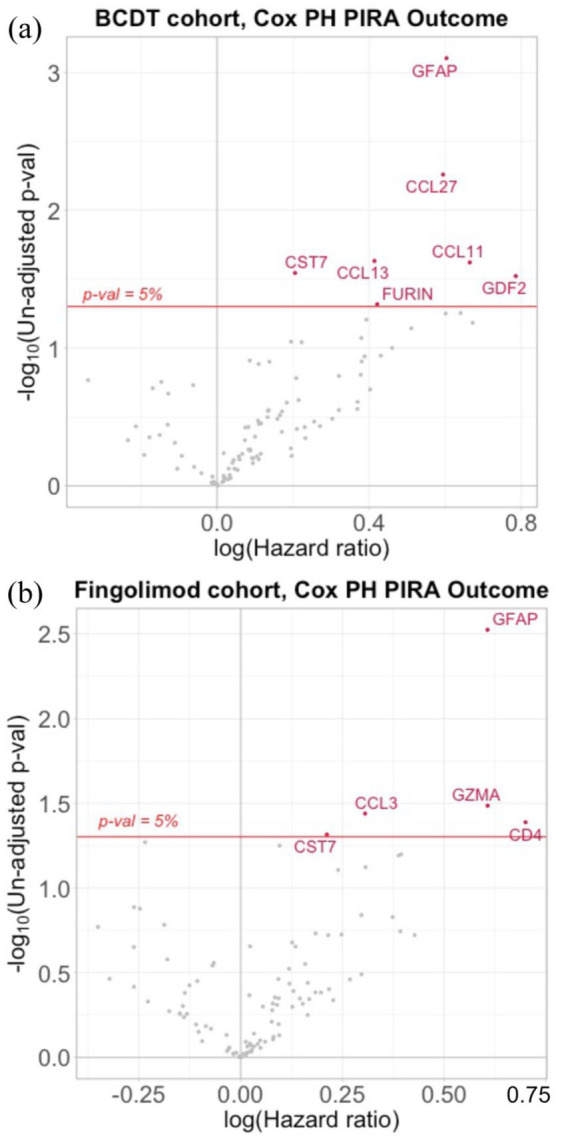
NULISA ‘Inflammation Panel’. Volcano plot of all proteins tested in NULISA ‘Inflammation Panel’ visualizing HR for PIRA prediction and statistical significance for (a) BCDT and (b) fingolimod cohort. Red dots indicate proteins predictive of subsequent PIRA, and grey dots proteins not reaching the un-adjusted p-value threshold of 0.05. The horizontal red line indicates the un-adjusted p-value threshold of 0.05. BCDT, B-cell depleting therapy; HR, hazard ratio; NULISA, NUcleic acid Linked Immuno-Sandwich Assay; PH, proportional-hazards; PIRA, progression independent of relapse activity.

Multivariable Cox regression models for time to PIRA identified NULISA ‘CNS Disease Panel’ derived GFAP (hazard ratio (HR) = 1.79, 95% confidence interval (CI) = 1.26–2.55, *p* = 0.0011, false discovery rate (FDR) *p* = 0.0745) and NfL (HR = 1.99, 95% CI = 1.31–3.02, *p* = 0.0013, FDR-adjusted *p* = 0.0745) as predictors of future PIRA (Figure 1(a)). Similarly, GFAP (HR = 1.83, 95% CI = 1.29–2.60, *p* = 0.0008, FDR-adjusted *p* = 0.0797) derived from the ‘Inflammation Panel’ was identified as predictor for future PIRA (Figure 2(a)). None of them passed adjustment for multiple comparisons (Table 2).

**Table 2.** Multivariable Cox regression models for time to PIRA, including NULISA targets with < 5% un-adjusted p-value.

Target	Events (= PIRA)	HR	95% CI	p-value un-adjusted	p-value FDR-adjusted
**‘CNS Disease Panel’**					
*BCDT cohort (n = 185)*					
GFAP	61	1.7942	1.2627;2.5494	0.0011	0.0745
NfL	61	1.9881	1.3096;3.0180	0.0013	0.0745
VGF	61	1.1498	1.0393;1.2720	0.0068	0.2511
CCL13	61	1.6022	1.1200;2.2919	0.0099	0.2511
TREM2	61	1.6967	1.1217;2.5664	0.0123	0.2511
IL12p70	61	0.6394	0.4498;0.9088	0.0127	0.2511
IL17A	61	1.1819	1.0236;1.3646	0.0227	0.3859
S100B	61	1.6584	1.0501;2.6191	0.0300	0.4465
*Fingolimod cohort (n = 200)*					
GFAP	69	1.7322	1.1377;2.6376	0.0104	0.9373
NPTX2	69	1.9056	1,0173;3,5696	0.0441	0.9373
**‘Inflammation Panel’**					
*BCDT cohort (n = 185)*					
GFAP	61	1.8278	1.2853;2.5993	0.0008	0.0797
CCL27	61	1.8117	1.1908;2.7563	0.0055	0.2783
CCL13	61	1.5123	1.0578;2.1621	0.0233	0.5059
CCL11	61	1.9434	1.0918;3.4590	0.0239	0.5059
CST7	61	1.2277	1.0218;1.4750	0.0285	0.5059
GDF2	61	2.1930	1.0787;4.4584	0.0301	0.5059
FURIN	61	1.5237	1.0036;2.3134	0.0481	0.5988
*Fingolimod cohort (n = 200)*					
GFAP	69	1.8341	1.2287;2.7377	0.0030	0.3029
GZMA	69	1.8348	1.0512;3.2026	0.0327	0.7193
CCL3	69	1.3576	1.0196;1.8078	0.0364	0.7193
CD4	69	2.0141	1.0291;3.9417	0.0410	0.7193
CST7	69	1.2359	1.0015;1.5252	0.0484	0.7193

Multivariable = adjusted for age, BMI and gender. BCDT, B cell-depleting therapy; CCL3, C-C motif chemokine 3; CCL11, C-C motif chemokine 11; CCL13, C-C motif chemokine 13; CCL27, C-C motif chemokine 27; CD4, T-cell surface glycoprotein CD4; CST7, cystatin-F; FDR, false discovery rate; FURIN, furin; GDF2, growth/differentiation factor; GFAP, glial fibrillary acidic protein; GZMA, granzyme A; HR, hazard ratio; IL12p70, interleukin-12 subunit beta|interleukin-12 subunit alpha; IL17A; interleukin 17A; NfL, neurofilament light chain; NPTX2, neuronal pentraxin-2; NULISA, NUcleic acid Linked Immuno-Sandwich Assay; PIRA, progression independent of relapse activity; S100B, S100 calcium-binding protein B; TREM2, triggering receptor expressed on myeloid cells 2; VGF, VGF nerve growth factor inducible.

**Figure 3.**
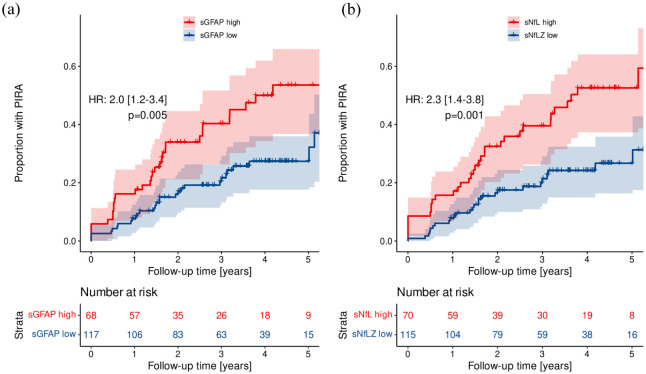
Time to PIRA after BCDT start. Kaplan–Meier curves showing the proportion of patients experiencing future PIRA when having ‘high’ (Z score > 1) versus ‘low’ (Z score ⩽ 1) biomarker levels (by Simoa) of sGFAP (a) and sNfL (b) at index sample. Patients with a ‘high’ sGFAP Z score at index sample (median 0.9 years after BCDT start) were at 2.0-fold risk of a future PIRA event versus those with ‘low’ sGFAP Z score (HR 2.0 (95% CI 1.2;3.4), *p* = 0.005); accordingly, patients with a ‘high’ sNfL Z score showed 2.3-fold increased risk to develop PIRA compared to patients with a ‘low’ sNfL Z score (HR 2.3 (95% CI 1.4;3.8), *p* = 0.001). BCDT, B cell-depleting therapy; HR, hazard ratio; PIRA, progression independent of relapse activity; sGFAP, serum glial fibrillary acidic protein; sNfL, serum neurofilament light chain.

By Simoa, ‘high’ Z score levels of sGFAP and sNfL for index samples were found to be prognostic for future PIRA (sGFAP: HR = 2.0 (95% CI = 1.2–3.4), *p* = 0.005, Figure 3(a); sNfL: HR 2.3 (95% CI = 1.4–3.8), *p* = 0.001, Figure 3(b)). When including absolute biomarker values in Cox models with outcome time to PIRA (Table 3), multivariable analysis for sGFAP (HR 1.66 per doubling of biomarker concentration (95% CI = 1.18–2.35), *p* = 0.004) and sNfL (HR = 1.59 (95% CI = 1.06–2.38), *p* = 0.024) were identified as predictors of future PIRA. In a multivariable model combining sGFAP and sNfL, the predictive value of sGFAP (HR = 1.52 (95% CI = 1.01–2.28), *p* = 0.045) persisted, while that of sNfL was attenuated (HR 1.24 (95% CI = 0.77–2.00), *p* = 0.385), potentially reflecting collinearity with sGFAP, rather than a complete absence of association.

**Table 3.** Multivariable Cox regression models with outcome time to PIRA.

Model	BCDT cohort (*n* = 185)			Fingolimod cohort (*n* = 200)		
	PIRA (*n* = 61 events)			PIRA (*n* = 69 events)		
	HR	95% CI	p-value	HR	95% CI	p-value
*Multivariable*						
sGFAP	1.66	1.18;2.35	**0.0040**	1.83	1.25;2.67	**0.0017**
age	1.03	1.01;1.05	**0.0067**	1.00	0.97;1.02	0.8927
BMI	0.95	0.89;1.01	0.1243	1.04	0.98;1.10	0.1895
Gender (woman)	0.72	0.42;1.24	0.2383	1.10	0.69;1.94	0.5850
*Multivariable*						
sNfL	1.59	1.06;2.38	**0.0238**	1.30	0.93;1.82	0.1187
age	1.02	1.00;1.05	0.0699	1.00	0.98;1.03	0.7005
BMI	0.96	0.90;1.02	0.2182	1.03	0.97;1.09	0.3234
Gender (woman)	0.79	0.47;1.35	0.3978	1.24	0.74;2.08	0.4188
*Multivariable*						
sGFAP	1.52	1.01;2.28	**0.0449**	1.76	1.18;2.63	**0.0052**
sNfL	1.24	0.77;2.00	0.3847	1.12	0.77;1.63	0.5508
age	1.02	1.00;1.05	0.0716	1.00	0.97;1.02	0.7843
BMI	0.96	0.90;1.02	0.2256	1.04	0.98;1.11	0.1556
Gender (woman)	0.71	0.41;1.22	0.2182	1.15	0.68;1.93	0.6022

Significant p-values are indicated in bold. BCDT, B cell-depleting treatment; BMI, body mass index; HR, hazard ratio; PIRA, progression independent of relapse activity; sGFAP, serum glial fibrillary acidic protein; sNfL, serum neurofilament light chain.

#### Fingolimod cohort

Using the NULISA ‘CNS Disease Panel’, GFAP (HR = 1.73, 95% CI = 1.14–2.64, *p* = 0.0104, FDR-adjusted *p* = 0.9373), but not NfL (HR = 1.25, 95% CI = 0.88–1.78, *p* = 0.2207, FDR-adjusted *p* = 0.9373), was identified as a predictor of future PIRA in the multivariable Cox regression model (Figure 1(b)). Similar, GFAP (HR = 1.83, 95% CI = 1.23–2.74, *p* = 0.0030, FDR-adjusted *p* = 0.3029) derived from the ‘Inflammation Panel’ was identified as a predictor for subsequent PIRA (Figure 2(b)). However, GFAP did not pass adjustment for multiple comparisons for either of the panels (Table 2).

**Figure 4.**
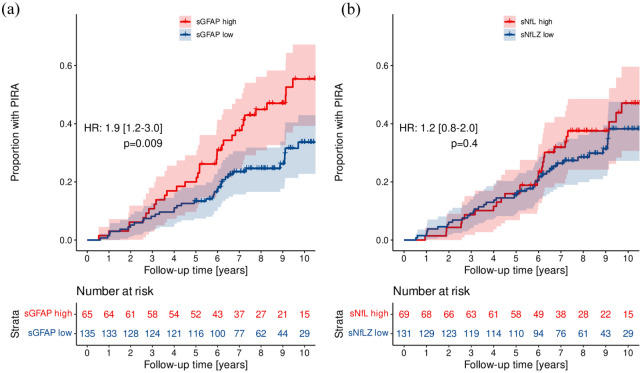
Time to PIRA after fingolimod start. Kaplan–Meier curves showing the proportion of patients experiencing future PIRA when having ‘high’ (sGFAP Z score > 0.75; sNfL Z score > 1) versus ‘low’ (sGFAP Z score ⩽ 0.75; sNfL Z score ⩽ 1) biomarker levels (by Simoa) of sGFAP (a) and sNfL (b) at index sample. Patients with a ‘high’ sGFAP Z score at index sample (median 1 year after fingolimod start) were at 1.9-fold risk of a future PIRA event versus those with a ‘low’ sGFAP Z score (HR 1.9 (95% CI 1.2;3.0), *p* = 0.009); accordingly, patients with a ‘high’ sNfL Z score showed no increased risk to develop PIRA compared to patients with a ‘low’ sNfL Z score (HR 1.2 (95% CI 0.8;2.0), *p* = 0.4). HR, hazard ratio; PIRA, progression independent of relapse activity; sGFAP, serum glial fibrillary acidic protein; sNfL, serum neurofilament light chain.

By Simoa ‘high’ Z score levels of sGFAP (HR = 1.9 (95% CI = 1.2–3.0), *p* = 0.009, Figure 4(a)), but not sNfL (HR = 1.2 (95% CI = 0.8–2.0), *p* = 0.4, Figure 4(b)), were identified as predictors of future PIRA. When absolute biomarker values were included in Cox models adjusted for age, BMI, and gender, analysing the time to PIRA, multivariable analysis indicated that sGFAP (HR = 1.83 (95% CI = 1.25–2.67), *p* = 0.002), but not sNfL (HR = 1.30 (95% CI = 0.93–1.82), *p* = 0.119) were significant predictors of future PIRA. This was confirmed when combining both biomarkers (sGFAP and sNfL) in a multivariable model (Table 3).

#### Correlation NULISA versus Simoa results

sGFAP (Simoa) correlated strongly with plasma GFAP NPQ units (NULISA ‘CNS Disease Panel’) in the BCDT cohort (GFAP: *r* = 0.90, *p* < 0.001) and fingolimod cohort (GFAP: *r* = 0.88, *p* < 0.001) (Figure S1A). This was confirmed with GFAP NPQ units obtained from the NULISA ‘Inflammation Panel’ (Figure S1B). Similarly, sNfL (Simoa) and NfL NPQ units (NULISA ‘CNS Disease Panel’) showed a strong correlation in both MS cohorts (BCDT: *r* = 0.92, *p* < 0.001; fingolimod: *r* = 0.92, *p* < 0.001) (Figure S1C).

#### Biomarkers beyond GFAP and NfL with potential to predict PIRA

##### The NULISA ‘CNS Disease Panel’

*BCDT cohort*. Multivariable Cox regression models for time to PIRA identified five additional NULISA targets (VGF nerve growth factor inducible (VGF), C-C motif chemokine 13 (CCL13), triggering receptor expressed on myeloid cells 2 (TREM2), interleukin 17A (IL17A), S100 calcium-binding protein *B* (S100B)) predictive for future PIRA. One target was identified as protective of future PIRA (interleukin-12 subunit beta|interleukin-12 subunit alpha (IL12p70)) (Figure 1(a)). The risk for future PIRA was strongest for VGF (HR = 1.15, 95% CI = 1.04–1.27, *p* = 0.0068, FDR-adjusted *p* = 0.2511) and CCL13 (HR = 1.60, 95% CI = 1.12–2.29, *p* = 0.0099, FDR-adjusted *p* = 0.2511). No target passed adjustment for multiple comparisons (Table 2).

*Fingolimod cohort*. One additional target (neuronal pentraxin-2 (NPTX2); HR = 1.9, 95% CI = 1.02–3.57, *p* = 0.0441, FDR-adjusted *p* = 0.9373) predictive of subsequent PIRA was identified (Figure 1(b)). NPTX2 did not remain significant after adjustment for multiple comparisons (Table 2).

##### The NULISA ‘Inflammation Panel’

*BCDT cohort*. Six targets (C-C motif chemokine 27 (CCL27), CCL13, C-C motif chemokine 11 (CCL11), cystatin-*F* (CST7), growth/differentiation factor 2 (GDF2), furin (FURIN)) were identified as predictive of subsequent PIRA in the multivariable Cox regression model (Figure 2(a)). Strongest association was seen for CCL27 (HR = 1.81, 95% CI = 1.19–2.76, *p* = 0.0055, FDR-adjusted *p* = 0.2783) and CCL13 (HR = 1.51, 95% CI = 1.06–2.16, *p* = 0.0233, FDR adjusted *p* = 0.5059). However, none of the targets remained significant after adjustment for multiple comparisons (Table 2).

*Fingolimod cohort*. Four targets (granzyme A (GZMA), C-C motif chemokine 3 (CCL3), T-cell surface glycoprotein CD4 (CD4), CST7) predictive of future PIRA were identified (Figure 2(b)). Strongest association was seen for GZMA (HR = 1.83, 95% CI = 1.05–3.20, *p* = 0.0327, FDR-adjusted *p* = 0.7193) and CCL3 (HR = 1.36, 95% CI = 1.02–1.81, *p* = 0.0364, FDR-adjusted *p* = 0.7193). No target passed correction for multiple comparisons (Table 2).

## Discussion

In this study, we performed NULISA proteomics on plasma samples 12 months after treatment initiation in BCDT and fingolimod treated pwMS, with available sGFAP and sNfL measurements (by Simoa) as comparison, and with a median follow-up of 4.0 (BCDT) and 9.1 (fingolimod) years. Among over 300 proteins investigated, GFAP was the main biomarker significantly related to time to PIRA in this cohort. We confirmed, by Simoa, sGFAP (sNfL only in BCDT cohort) as a promising biomarker for capturing and predicting disease progression in pwMS.

sGFAP and sNfL are linked to disease progression (i.e. PIRA) in MS, with sNfL also associated with focal inflammatory disease activity (i.e. relapse-associated worsening (RAW)).^4–8,15–17^ As current disease-modifying therapies mainly and successfully target mechanisms underlying RAW, the goal of future therapeutic approaches is to prevent PIRA and to discover biomarkers to capture and forecast it. GFAP is thought to reflect the degree of astrocyte activation or astrogliosis (i.e. a complex, multifaceted response of astrocytes to damage and disease in the CNS), as astrogliosis is characterized by rapid release of GFAP.^18^ Thus, GFAP is theoretically a potential biomarker of disease progression, as confirmed in several studies,^4–6,8,15–17,19^ including present results, although not in all.^20,21^

Early prognosis prediction in MS is crucial for optimizing treatment strategies and balancing benefit-risk factors. We utilized a highly sensitive and robust proteomic platform (NULISA) to conduct targeted analysis of 322 unique plasma proteins of 385 pwMS. Fourteen proteins predictive of future PIRA were identified. Of these, GFAP and NfL are promising or established as important indicators for prognostic evaluation and treatment outcomes in pwMS.^4–7,15,16,19,22,23^ The protein GZMA is less well recognized as prognostic marker,^24^ while TREM2,^25^ IL17A,^26^ S100B,^27^ and CD4 are studied for their role in MS pathogenesis and as possible therapeutic targets, and the other identified proteins (VGF, CCL13, NPTX2, CCL11, CST7, GDF2, FURIN) limited or not at all studied in MS. None of these identified proteins remained predictive of PIRA after multiple testing corrections. However, GFAP consistently showed the highest hazard for prediction of future disease progression across panels and cohorts. Importantly, a biomarker for progression risk could guide early high-efficacy treatment, which improves long-term outcomes.^28^

We failed to identify any other promising biomarker to predict PIRA, apart from the already highly promising GFAP. ^4–6,15,16,19^ This is noteworthy, as over 300 proteins focusing on neurodegenerative diseases, cytokines, and chemokines associated with inflammation and immune responses were analysed. This could be because disease-modifying treatments alter the proteome, masking potential blood signatures of progressive biology. Furthermore, predicting an unpredictable disease and a difficult-to-measure outcome like PIRA presents significant challenges. However, again GFAP did, making the GFAP results even more remarkable. Maybe a more approachable way is to measure longitudinal samples during progression instead of trying to prognosticate PIRA.^4–6^

We found consistently higher GFAP Z scores in pwMS experiencing PIRA, compared to those without. That future PIRA can be determined from a single GFAP measurement in an accessible compartment such as blood,^4,5^ confirmed in present study, has several advantages, including enabling early detection of pwMS at higher risk of future PIRA, guide treatment decisions, and sampling being minimally invasive. Furthermore, these features render sGFAP a potentially valuable biomarker in selecting patients for clinical trials targeting disease progression.

Simoa (serum) and NULISA (plasma) results showed high correlations for GFAP and NfL. A significant correlation between serum, plasma, and also cerebrospinal fluid (CSF) GFAP and NfL is shown previously.^29,30^ Consistent results were observed for NULISA derived GFAP across both the ‘CNS Disease Panel’ and ‘Inflammation Panel’ (NfL only included in the ‘CNS Disease Panel’), as well as between the two cohorts of pwMS. The correlation of GFAP between various biological matrices, including serum, plasma, and CSF, the consistent results between the two NULISA panels and the both pwMS cohorts, and with the NULISA GFAP measurements showing a good correlation to single-plex methods, highlights the reliability of GFAP and the innovative multiplex measurement approach.

New high-plex solutions with attomolar sensitivity, like Olink, SomaLogic, and NULISA, provide targeted screening of hundreds to thousands of proteins, using low volumes and streamlined workflows. However, there are limited data on these platforms’ potential within the MS field, although some Olink studies exist. One report shared immunologic mechanism in both relapsing and progressive MS, while emphasizing the significant impact of ageing on the intrathecal immune response,^31^ but the sample size was small, why the results should be treated with caution. Another study reported a set of 11 CSF proteins important for prediction of disability outcome measured with normalized age-related MS score (nARMSS), but in plasma only NfL was predictive of nARMSS.^32^ Again, the sample size was limited and the follow-up time varied. A third study utilizing UK Biobank data identified 72 MS associated proteins, including novel findings such as an MS-specific reduction of GZMA, however the MS cohort was not well-characterized.^33^ Olink is generally less sensitive for low-abundance proteins compared to NULISA.^9^ To our knowledge, NULISA has not been used in long-term, well-characterized MS cohorts as in this study, though some applications in dementia have been reported.^30^ Highlighting the feasibility and potential of immunoassay-based multiplexing to provide a comprehensive view of proteomic changes, and offering a blood-based diagnostic and prognostic tool for unaddressed needs in neurodegenerative disorders.

Our study comes with limitations. First, we lacked a comparison group to evaluate the potential treatment effect of BCDT and fingolimod on targeted biomarker levels, such as untreated individuals with MS. Second, the results may not be generalizable to a universal MS population as this study included patients eligible for BCDT and fingolimod, and no other treatments. However, most patients had previously been exposed to other disease-modifying therapies, preferably low-efficacy treatments, before entering this study. Third, our methods for clinically evaluating PIRA lack accuracy. However, PIRA is increasingly recognized as a crucial outcome measure in MS, and efforts are being made to harmonize the definitions of PIRA and to improve comparability across studies.^1^ Finally, a limitation of this study was the limited sample size compared to the large number of targets assessed, which limited the statistical power to detect moderate effect sizes after correcting for multiple testing. Given the exploratory nature of the work, the primary aim was to identify potential candidates rather than establish definitive associations, the analysis was therefore primarily qualitative and in comparison to previously identified promising blood biomarkers in MS.

In conclusion, among the over 300 proteins analysed using NULISA in two different cohorts, GFAP was the main biomarker significantly associated with risk of PIRA. Furthermore, our findings confirm, through Simoa, that sGFAP is a promising biomarker for detecting and predicting disease progression in pwMS.

## Supplemental Material

sj-docx-1-msj-10.1177_13524585251375780 – Supplemental material for Biomarker discovery using NUcleic Acid-Linked Immuno-Sandwich Assay in multiple sclerosis patients experiencing progression independent of relapse activity Supplemental material, sj-docx-1-msj-10.1177_13524585251375780 for Biomarker discovery using NUcleic Acid-Linked Immuno-Sandwich Assay in multiple sclerosis patients experiencing progression independent of relapse activity by Sofia Sandgren, Aleksandra Maleska Maceski, Pascal Benkert, Maximilian Einsiedler, Sabine Schaedelin, Johanna Oechtering, Lutz Achtnichts, Patrice H Lalive, Stefanie Müller, Caroline Pot, Amanda Heslegrave, David Hunt, Jan Lycke, Robert Hoepner, Patrick Roth, Claudio Gobbi, Manuel Comabella, Tobias Derfuss, Ludwig Kappos, Cristina Granziera, Ahmed Abdelhak, David Leppert, Eline AJ Willemse, Henrik Zetterberg and Jens Kuhle in Multiple Sclerosis Journal
