# Supplementary material for: Biomarker discovery using NUcleic Acid-Linked Immuno-Sandwich Assay in multiple sclerosis patients experiencing progression independent of relapse activity
Source: Mult Scler. 2025 Oct 14;31(13):1531–42. doi: 10.1177/13524585251375780 (PMC12589673; doi:10.1177/13524585251375780)
Supplement: sj-docx-1-msj-10.1177_13524585251375780 – Supplemental material for Biomarker discovery using NUcleic Acid-Linked Immuno-Sandwich Assay in multiple sclerosis patients experiencing progression independent of relapse activity [file sj-docx-1-msj-10.1177_13524585251375780.docx]

Supplementary Material

**Biomarker discovery using NUcleic Acid-Linked Immuno-Sandwich Assay in multiple sclerosis patients experiencing progression independent of relapse activity**

Sofia Sandgren*, Aleksandra Maleska Maceski*, Pascal Benkert et al.

* Shared first authorship

Index Page

**Supplementary Methods** 2-4

**Supplementary Tables**

**Table S1** Targets NULISA ‘CNS Disease Panel’ 120 4-9

**Table S2** Targets NULISA ‘Inflammation Panel’ 250 9-20

**Table S3** Overlapping targets between NULISA ‘CNS Disease

Panel’ 120 and ‘Inflammation Panel’ 250 20-22

**Supplementary Figures**

**Fig S1** Correlation Simoa versus NULISA results. 23

**Supplementary References** 24

## **Supplementary Methods**

*The Swiss Multiple Sclerosis Cohort*

The Swiss Multiple Sclerosis Cohort (SMSC; NCT02433028) is a prospective multicentre cohort study performed across eight Swiss academic medical centres.^1, 2^ Demographic, neuroimaging, and clinical data as well as blood samples are collected every 6 or 12 months, and stored at -80 °C according to standardized procedures.^3^ Standardized clinical assessments with the Expanded Disability Status Scale (EDSS)^4^ score calculations are performed by Neurostatus e-Test certified raters.^5^

*Clinical measures: disability worsening and PIRA*

A relapse was defined as an episode of new, worsening or recurrent neurological disturbance lasting for at least 24 h.^6^ Due to the observational study design, a roving baseline approach (*i.e.*, the reference EDSS score is updated over time rather than fixed at baseline) was applied.^7^ The reference was reset after a ≥6 months confirmed EDSS improvement (re-baselining).

*NUcleic acid Linked Immuno-Sandwich Assay (NULISA) analysis*

The NULISA assays were conducted at Alamar Biosciences as previously outlined.^8^ In summary, Ethylenediaminetetraacetic acid plasma samples were stored at -80°C. Prior to the assay, samples were thawed and centrifuged at 10,000g for 10 minutes. A volume of 10 μL of the supernatant was then added to 96-well plates and analyzed using the ‘CNS Disease Panel’ 120 (124 targets), which primarily targets protein markers related to neurodegenerative diseases, and ‘Inflammation Panel’ 250 (250 targets), targeting mostly inflammation and immune response-related cytokines and chemokines. A total of 52 proteins were shared between the two panels, resulting in the examination of 322 unique proteins. A list of all targets included in the ‘CNS Disease Panel’, the ‘Inflammation Panel’, and overlapping proteins is provided in Table S1, S2, and S3. Assay controls (sample controls [SCs]: three replicates; internal-plate controls [IPCs]: three replicates; negative controls [NCs]: four replicates) were included per plate. The performance of the SCs included in the ‘CNS Disease Panel’ exhibited an intra-plate median coefficient of variation (CV) of 6.14%, and an inter-plate CV of 7.4%. For the ‘Inflammation Panel’, the intra-plate median CV was 6.0%, while the inter-plate CV was 7.8%. Inter-plate normalization was performed using the median count data from target-specific IPCs across plates, thereby adjusting for potential run-to-run and inter-plate variability, as previously described.^8^ Detectability (*i.e.*, the % of targets quantified above the Limit of Detection [LOD] in >50% of the sample cohort) was 96.0% for the ‘CNS Disease Panel’ and 99.6% for the ‘Inflammation Panel’. The LOD was calculated separately with attomolar level sensitivity for each target using NCs samples.^8^ Samples with signals below the LOD were assigned their original values and included in the analyses, except for CV calculations where they were excluded.

The NULISA workflow was carried out using an automated instrument from Hamilton. Briefly, immunocomplexes were formed with paired oligo-conjugated antibodies, followed by initial capture with oligo-dT beads, release, secondary capture with streptavidin beads, and finally ligation to generate a DNA reporter molecule containing both target-specific and sample-specific barcodes. DNA reporter molecules were then pooled, amplified using PCR, purified, and sequenced on the Illumina NextSeq 2000.

The NULISA algorithm (Alamar Biosciences) was used to process sequenced data for NULISA. The target-specific and sample-specific barcodes were quantified, and up to two mismatching bases or one indel and one mismatch were allowed. Internal control normalization was done by dividing target counts for each sample by that sample’s internal control counts. Inter-plate normalization was then performed using IPC normalization, wherein counts were divided by target-specific medians of the three IPC wells on that plate. Data were then rescaled, and log2-transformed to obtain NULISA Protein Quantification (NPQ) units for downstream statistical analysis.

## **Table S1** Targets NULISA ‘CNS Disease Panel’ 120 (n=124).

| **Target** | **Protein ID** | **Protein name** |
| --- | --- | --- |
| ACHE | P22303 | Acetylcholinesterase |
| AGRN | O00468 | Agrin |
| ANXA5 | P08758 | Annexin A5 |
| APOE | P02649 | Apolipoprotein E |
| APOE4 | P02649 | Apolipoprotein E |
| ARSA | P15289 | Arylsulfatase A |
| Ab38 | P05067 | Amyloid-beta precursor protein |
| Ab40 | P05067 | Amyloid-beta precursor protein |
| Ab42 | P05067 | Amyloid-beta precursor protein |
| BACE1 | P56817 | Beta-secretase 1 |
| BASP1 | P80723 | Brain abundant membrane attached signal protein 1 |
| BDNF | P23560 | Brain-derived neurotrophic factor |
| CALB2 | P22676 | Calretinin |
| CCL11 | P51671 | Eotaxin |
| CCL13 | Q99616 | C-C motif chemokine 13 |
| CCL17 | Q92583 | C-C motif chemokine 17 |
| CCL2 | P13500 | C-C motif chemokine 2 |
| CCL22 | O00626 | C-C motif chemokine 22 |
| CCL26 | Q9Y258 | C-C motif chemokine 26 |
| CCL3 | P10147 | C-C motif chemokine 3 |
| CCL4 | P13236 | C-C motif chemokine ligand 4 |
| CD40LG | P29965 | CD40 ligand |
| CD63 | P08962 | CD63 antigen |
| CHI3L1 | P36222 | Chitinase-3-like protein 1 |
| CHIT1 | Q13231 | Chitotriosidase-1 |
| CNTN2 | Q02246 | Contactin-2 |
| CRH | P06850 | Corticoliberin |
| CRP | P02741 | C-reactive protein |
| CSF2 | P04141 | Granulocyte-macrophage colony-stimulating factor |
| CST3 | P01034 | Cystatin-C |
| CX3CL1 | P78423 | Fractalkine |
| CXCL1 | P09341 | Growth-regulated alpha protein |
| CXCL10 | P02778 | C-X-C motif chemokine 10 |
| CXCL8 | P10145 | Interleukin-8, IL8 |
| ENO2 | P09104 | Gamma-enolase |
| FABP3 | P05413 | Fatty acid-binding protein, heart |
| FCN2 | Q15485 | Ficolin-2 |
| FGF2 | P09038 | Fibroblast growth factor 2 |
| FLT1 | P17948 | Vascular endothelial growth factor receptor 1 |
| FOLR1 | P15328 | Folate receptor alpha |
| GDF15 | Q99988 | Growth/differentiation factor 15 |
| GDI1 | P31150 | Rab GDP dissociation inhibitor alpha |
| GDNF | P39905 | Glial cell line-derived neurotrophic factor |
| GFAP | P14136 | Glial fibrillary acidic protein |
| GOT1 | P17174 | Aspartate aminotransferase, cytoplasmic |
| HBA1 | P69905 | Hemoglobin subunit alpha |
| HTT | P42858 | Huntingtin |
| ICAM1 | P05362 | Intercellular adhesion molecule 1 |
| IFNG | P01579 | Interferon gamma |
| IGF1R | P08069 | Insulin-like growth factor 1 receptor |
| IGFBP7 | Q16270 | Insulin-like growth factor-binding protein 7 |
| IL10 | P22301 | Interleukin-10 |
| IL12p70 | P29459\|P29460 | Interleukin-12 subunit beta\|Interleukin-12 subunit alpha |
| IL13 | P35225 | Interleukin-13 |
| IL15 | P40933 | Interleukin-15 |
| IL16 | Q14005 | Pro-interleukin-16 |
| IL17A | Q16552 | Interleukin-17A |
| IL18 | Q14116 | Interleukin-18 |
| IL1B | P01584 | Interleukin-1 beta |
| IL2 | P60568 | Interleukin-2 |
| IL33 | O95760 | Interleukin 33 |
| IL4 | P05112 | Interleukin-4 |
| IL5 | P05113 | Interleukin-5 |
| IL6 | P05231 | Interleukin-6 |
| IL6R | P08887 | Interleukin-6 receptor subunit alpha |
| IL7 | P13232 | Interleukin-7 |
| IL9 | P15248 | Interleukin-9 |
| KDR | P35968 | Vascular endothelial growth factor receptor 2 |
| KLK6 | Q92876 | Kallikrein-6 |
| MAPT | P10636 | Microtubule-associated protein tau |
| MDH1 | P40925 | Malate dehydrogenase, cytoplasmic |
| MME | P08473 | Membrane metalloendopeptidase |
| MSLN | Q13421 | Mesothelin |
| NEFH | P12036 | Neurofilament heavy polypeptide |
| NEFL | P07196 | Neurofilament light polypeptide |
| NGF | P01138 | Beta-nerve growth factor |
| NPTX1 | Q15818 | Neuronal pentraxin-1 |
| NPTX2 | P47972 | Neuronal pentraxin-2 |
| NPTXR | O95502 | Neuronal pentraxin receptor |
| NPY | P01303 | Neuropeptide Y |
| NRGN | Q92686 | Neurogranin |
| Oligo-SNCA | P37840 | Alpha-synuclein |
| PARK7 | Q99497 | Protein/nucleic acid deglycase DJ-1 |
| PDGFRB | P09619 | Platelet-derived growth factor receptor beta |
| PDLIM5 | Q96HC4 | PDZ and LIM domain 5 |
| PGF | P49763 | Placenta growth factor |
| PGK1 | P00558 | Phosphoglycerate kinase 1 |
| POSTN | Q15063 | Periostin |
| PRDX6 | P30041 | Peroxiredoxin-6 |
| pSNCA-129 | P37840 | Alpha-synuclein |
| PSEN1 | P49768 | Presenilin 1 |
| pTau-181 | P10636 | Microtubule-associated protein tau |
| pTau-217 | P10636 | Microtubule-associated protein tau |
| pTau-231 | P10636 | Microtubule-associated protein tau |
| pTDP43-409 | Q13148 | TAR DNA-binding protein 43 |
| PTN | P21246 | Pleiotrophin |
| REST | Q13127 | RE1 silencing transcription factor |
| RUVBL2 | Q9Y230 | RuvB like AAA ATPase 2 |
| S100A12 | P80511 | Protein S100-A12 |
| S100B | P04271 | S100 calcium binding protein B |
| SAA1 | P0DJI8 | Serum amyloid A-1 protein |
| SFRP1 | Q8N474 | Secreted frizzled-related protein 1 |
| SFTPD | P35247 | Pulmonary surfactant-associated protein D |
| SLIT2 | O94813 | Slit homolog 2 protein |
| SMOC1 | Q9H4F8 | SPARC-related modular calcium-binding protein 1 |
| SNAP25 | P60880 | Synaptosomal-associated protein 25 |
| SNCA | P37840 | Alpha-synuclein |
| SNCB | Q16143 | Synuclein beta |
| SOD1 | P00441 | Superoxide dismutase [Cu-Zn] |
| SQSTM1 | Q13501 | Sequestosome-1 |
| TAFA5 | Q7Z5A7 | Chemokine-like protein TAFA-5 |
| TARDBP | Q13148 | TAR DNA-binding protein 43 |
| TEK | Q02763 | Angiopoietin-1 receptor |
| TIMP3 | P35625 | Metalloproteinase inhibitor 3 |
| TNF | P01375 | Tumor necrosis factor |
| TREM1 | Q9NP99 | Triggering receptor expressed on myeloid cells 1 |
| TREM2 | Q9NZC2 | Triggering receptor expressed on myeloid cells 2 |
| UCHL1 | P09936 | Ubiquitin carboxyl-terminal hydrolase isozyme L1 |
| VCAM1 | P19320 | Vascular cell adhesion protein 1 |
| VEGFA | P15692 | Vascular endothelial growth factor A |
| VEGFD | O43915 | Vascular endothelial growth factor D |
| VGF | O15240 | VGF nerve growth factor inducible |
| VSNL1 | P62760 | Visinin-like protein 1 |
| YWHAZ | P63104 | 14-3-3 protein zeta/delta |

Targets separated by a line (|) means that the antibody pair used detects the complex containing both subunits.

## **Table S2** Targets NULISA ‘Inflammation Panel’ 250 (n=250).

| **Target** | **Protein ID** | **Protein name** |
| --- | --- | --- |
| AGER | Q15109 | Advanced glycosylation end product-specific receptor |
| AGRP | O00253 | Agouti-related protein |
| ANGPT1 | Q15389 | Angiopoietin-1 |
| ANGPT2 | O15123 | Angiopoietin-2 |
| ANXA1 | P04083 | Annexin A1 |
| AREG | P15514 | Amphiregulin |
| BDNF | P23560 | Brain-derived neurotrophic factor |
| BMP7 | P18075 | Bone morphogenetic protein 7 |
| BST2 | Q10589 | Bone marrow stromal antigen 2 |
| C1QA | P02745 | Complement C1q A chain |
| CALCA | P01258 | Calcitonin [Cleaved into: Calcitonin; Katacalcin |
| CCL1 | P22362 | C-C motif chemokine 1 |
| CCL11 | P51671 | Eotaxin |
| CCL13 | Q99616 | C-C motif chemokine 13 |
| CCL14 | Q16627 | C-C motif chemokine 14 |
| CCL15 | Q16663 | C-C motif chemokine 15 |
| CCL16 | O15467 | C-C motif chemokine 16 |
| CCL17 | Q92583 | C-C motif chemokine 17 |
| CCL19 | Q99731 | C-C motif chemokine 19 |
| CCL2 | P13500 | C-C motif chemokine 2 |
| CCL20 | P78556 | C-C motif chemokine 20 |
| CCL21 | O00585 | C-C motif chemokine 21 |
| CCL22 | O00626 | C-C motif chemokine 22 |
| CCL23 | P55773 | C-C motif chemokine 23 |
| CCL24 | O00175 | C-C motif chemokine 24 |
| CCL25 | O15444 | C-C motif chemokine 25 |
| CCL26 | Q9Y258 | C-C motif chemokine 26 |
| CCL27 | Q9Y4X3 | C-C motif chemokine 27 |
| CCL28 | Q9NRJ3 | C-C motif chemokine 28 |
| CCL3 | P10147 | C-C motif chemokine 3 |
| CCL4 | P13236 | C-C motif chemokine ligand 4 |
| CCL5 | P13501 | C-C motif chemokine 5 |
| CCL7 | P80098 | C-C motif chemokine 7 |
| CCL8 | P80075 | C-C motif chemokine 8 |
| CD200 | P41217 | OX-2 membrane glycoprotein |
| CD200R1 | Q8TD46 | Cell surface glycoprotein CD200 receptor 1 |
| CD27 | P26842 | CD27 antigen |
| CD274 | Q9NZQ7 | Programmed cell death 1 ligand 1, PD-L1 |
| CD276 | Q5ZPR3 | CD276 molecule |
| CD3E | P07766 | T-cell surface glycoprotein CD3 epsilon chain |
| CD4 | P01730 | T-cell surface glycoprotein CD4 |
| CD40 | P25942 | CD40 molecule |
| CD40LG | P29965 | CD40 ligand |
| CD46 | P15529 | Membrane cofactor protein |
| CD70 | P32970 | CD70 antigen |
| CD80 | P33681 | T-lymphocyte activation antigen CD80 |
| CD83 | Q01151 | CD83 antigen |
| CD93 | Q9NPY3 | Complement component C1q receptor |
| CEACAM5 | P06731 | Carcinoembryonic antigen related cell adhesion molecule 5 |
| CHI3L1 | P36222 | Chitinase-3-like protein 1 |
| CLEC4A | Q9UMR7 | C-type lectin domain family 4 member A |
| CNTF | P26441 | Ciliary neurotrophic factor |
| CRP | P02741 | C-reactive protein |
| CSF1 | P09603 | Macrophage colony-stimulating factor 1 |
| CSF1R | P07333 | Macrophage colony-stimulating factor 1 receptor |
| CSF2 | P04141 | Granulocyte-macrophage colony-stimulating factor |
| CSF2RB | P32927 | Cytokine receptor common subunit beta |
| CSF3 | P09919 | Granulocyte colony-stimulating factor, G-CSF |
| CSF3R | Q99062 | Granulocyte colony-stimulating factor receptor |
| CST7 | O76096 | Cystatin-F |
| CTF1 | Q16619 | Cardiotrophin-1 |
| CTLA4 | P16410 | Cytotoxic T-lymphocyte protein 4 |
| CTSS | P25774 | Cathepsin S |
| CX3CL1 | P78423 | Fractalkine |
| CXADR | P78310 | Coxsackievirus and adenovirus receptor |
| CXCL1 | P09341 | Growth-regulated alpha protein |
| CXCL10 | P02778 | C-X-C motif chemokine 10 |
| CXCL11 | O14625 | C-X-C motif chemokine 11 |
| CXCL12 | P48061 | Stromal cell-derived factor 1 |
| CXCL13 | O43927 | C-X-C motif chemokine 13 |
| CXCL14 | O95715 | C-X-C motif chemokine 14 |
| CXCL16 | Q9H2A7 | C-X-C motif chemokine 16 |
| CXCL2 | P19875 | C-X-C motif chemokine ligand 2 |
| CXCL3 | P19876 | C-X-C motif chemokine ligand 3 |
| CXCL5 | P42830 | C-X-C motif chemokine 5 |
| CXCL6 | P80162 | C-X-C motif chemokine 6 |
| CXCL8 | P10145 | Interleukin-8, IL8 |
| CXCL9 | Q07325 | C-X-C motif chemokine 9 |
| EGF | P01133 | Pro-epidermal growth factor |
| EPO | P01588 | Erythropoietin |
| FASLG | P48023 | Tumor necrosis factor ligand superfamily member 6 |
| FGF19 | O95750 | Fibroblast growth factor 19 |
| FGF2 | P09038 | Fibroblast growth factor 2 |
| FGF21 | Q9NSA1 | Fibroblast growth factor 21 |
| FGF23 | Q9GZV9 | Fibroblast growth factor 23 |
| FLT1 | P17948 | Vascular endothelial growth factor receptor 1 |
| FLT3LG | P49771 | Fms-related tyrosine kinase 3 ligand |
| FLT4 | P35916 | Vascular endothelial growth factor receptor 3 |
| FTH1 | P02794 | Ferritin heavy chain 1 |
| FURIN | P09958 | Furin |
| GDF15 | Q99988 | Growth/differentiation factor 15 |
| GDF2 | Q9UK05 | Growth/differentiation factor 2 |
| GFAP | P14136 | Glial fibrillary acidic protein |
| GRN | P28799 | Progranulin |
| GZMA | P12544 | Granzyme A |
| GZMB | P10144 | Granzyme B |
| HAVCR1 | Q96D42 | Hepatitis A virus cellular receptor 1 |
| HGF | P14210 | Hepatocyte growth factor |
| HLA-DRA | P01903 | Major histocompatibility complex, class II, DR alpha |
| ICAM1 | P05362 | Intercellular adhesion molecule 1 |
| ICOSLG | O75144 | ICOS ligand |
| IFNA1; IFNA13 | P01562; P01562 | Interferon alpha-1; Interferon alpha-13 |
| IFNA2 | P01563 | Interferon alpha-2 |
| IFNB1 | P01574 | Interferon beta |
| IFNG | P01579 | Interferon gamma |
| IFNL1 | Q8IU54 | Interferon lambda-1 |
| IFNW1 | P05000 | Interferon omega-1 |
| IKBKG | Q9Y6K9 | Inhibitor of nuclear factor kappa B kinase subunit gamma |
| IL10 | P22301 | Interleukin-10 |
| IL10RB | Q08334 | Interleukin-10 receptor subunit beta |
| IL11 | P20809 | Interleukin-11 |
| IL12B | P29460 | Interleukin-12 subunit beta |
| IL12p70 | P29459\|P29460 | Interleukin-12 subunit beta\|Interleukin-12 subunit alpha |
| IL12RB1 | P42701 | Interleukin-12 receptor subunit beta-1 |
| IL13 | P35225 | Interleukin-13 |
| IL13RA2 | Q14627 | Interleukin 13 receptor subunit alpha 2 |
| IL15 | P40933 | Interleukin-15 |
| IL15RA | Q13261 | Interleukin-15 receptor subunit alpha |
| IL16 | Q14005 | Pro-interleukin-16 |
| IL17A | Q16552 | Interleukin-17A |
| IL17A\|IL17F | Q16552\|Q96PD4 | Interleukin-17A\|Interleukin-17F |
| IL17B | Q9UHF5 | Interleukin-17B |
| IL17C | Q9P0M4 | Interleukin-17C |
| IL17F | Q96PD4 | Interleukin-17F |
| IL17RA | Q96F46 | Interleukin-17 receptor A |
| IL17RB | Q9NRM6 | Interleukin-17 receptor B |
| IL18 | Q14116 | Interleukin-18 |
| IL18BP | O95998 | Interleukin-18-binding protein |
| IL18R1 | Q13478 | Interleukin-18 receptor 1 |
| IL19 | Q9UHD0 | Interleukin-19 |
| IL1B | P01584 | Interleukin-1 beta |
| IL1R1 | P14778 | Interleukin-1 receptor type 1 |
| IL1R2 | P27930 | Interleukin-1 receptor type 2 |
| IL1RL1 | Q01638 | Interleukin-1 receptor-like 1 |
| IL1RN | P18510 | Interleukin-1 receptor antagonist protein |
| IL2 | P60568 | Interleukin-2 |
| IL20 | Q9NYY1 | Interleukin-20 |
| IL22 | Q9GZX6 | Interleukin-22 |
| IL23 | P29460\|Q9NPF7 | Interleukin-12 subunit beta\|Interleukin-23 subunit alpha |
| IL24 | Q13007 | Interleukin-24 |
| IL27 | Q8NEV9\|Q14213 | Interleukin 27\|Interleukin-27 subunit beta |
| IFNL2; IFNL3 | Q8IZJ0; Q8IZI9 | Interferon lambda-2; Interferon lambda-3 |
| IL2RA | P01589 | Interleukin-2 receptor subunit alpha |
| IL2RB | P14784 | Interleukin-2 receptor subunit beta |
| IL32 | P24001 | Interleukin-32 |
| IL33 | O95760 | Interleukin 33 |
| IL34 | Q6ZMJ4 | Interleukin-34 |
| IL36A | Q9UHA7 | Interleukin-36 alpha |
| IL36B | Q9NZH7 | Interleukin-36 beta |
| IL36G | Q9NZH8 | Interleukin-36 gamma |
| IL3RA | P26951 | Interleukin-3 receptor subunit alpha |
| IL4 | P05112 | Interleukin-4 |
| IL4R | P24394 | Interleukin-4 receptor subunit alpha |
| IL5 | P05113 | Interleukin-5 |
| IL5RA | Q01344 | Interleukin-5 receptor subunit alpha |
| IL6 | P05231 | Interleukin-6 |
| IL6R | P08887 | Interleukin-6 receptor subunit alpha |
| IL6ST | P40189 | Interleukin-6 receptor subunit beta |
| IL7 | P13232 | Interleukin-7 |
| IL7R | P16871 | Interleukin-7 receptor subunit alpha |
| IL9 | P15248 | Interleukin-9 |
| IRAK4 | Q9NWZ3 | Interleukin-1 receptor-associated kinase 4 |
| KDR | P35968 | Vascular endothelial growth factor receptor 2 |
| KITLG | P21583 | Kit ligand |
| KLRK1 | P26718 | NKG2-D type II integral membrane protein |
| KNG1 | P01042 | Kininogen-1 |
| LAG3 | P18627 | Lymphocyte activation gene 3 protein |
| LAMP3 | Q9UQV4 | Lysosomal associated membrane protein 3 |
| LCN2 | P80188 | Neutrophil gelatinase-associated lipocalin |
| LGALS9 | O00182 | Galectin-9 |
| LIF | P15018 | Leukemia inhibitory factor |
| LILRB2 | Q8N423 | Leukocyte immunoglobulin-like receptor subfamily B member 2 |
| LTA | P01374 | Lymphotoxin-alpha |
| LTA\|LTB | P01374\|Q06643 | Lymphotoxin-alpha\|Lymphotoxin-beta |
| MERTK | Q12866 | MER proto-oncogene, tyrosine kinase |
| MICA | Q29983 | MHC class I polypeptide-related sequence A |
| MICB | Q29980 | MHC class I polypeptide-related sequence B |
| MIF | P14174 | Macrophage migration inhibitory factor |
| MMP1 | P03956 | Interstitial collagenase |
| MMP12 | P39900 | Macrophage metalloelastase |
| MMP3 | P08254 | Stromelysin-1 |
| MMP8 | P22894 | Neutrophil collagenase |
| MMP9 | P14780 | Matrix metalloproteinase-9 |
| MPO | P05164 | Myeloperoxidase |
| MUC16 | Q8WXI7 | Mucin-16 |
| NAMPT | P43490 | Nicotinamide phosphoribosyltransferase |
| NCR1 | O76036 | Natural cytotoxicity triggering receptor 1 |
| NGF | P01138 | Beta-nerve growth factor |
| NTF3 | P20783 | Neurotrophin-3 |
| OSM | P13725 | Oncostatin-M |
| OSMR | Q99650 | Oncostatin-M-specific receptor subunit beta |
| PDCD1 | Q15116 | Programmed cell death protein 1 |
| PDCD1LG2 | Q9BQ51 | Programmed cell death 1 ligand 2 |
| PDGFA | P04085 | Platelet-derived growth factor subunit A |
| PDGFB | P01127 | Platelet-derived growth factor subunit B |
| PTX3 | P26022 | Pentraxin-related protein PTX3 |
| S100A12 | P80511 | Protein S100-A12 |
| S100A9 | P06702 | Protein S100-A9 |
| SCG2 | P13521 | Secretogranin II |
| SDC1 | P18827 | Syndecan 1 |
| SELE | P16581 | E-selectin |
| SELP | P16109 | P-selectin |
| SIRPA | P78324 | Tyrosine-protein phosphatase non-receptor type substrate 1 |
| SLAMF1 | Q13291 | Signaling lymphocytic activation molecule |
| SPP1 | P10451 | Osteopontin |
| TAFA5 | Q7Z5A7 | Chemokine-like protein TAFA-5 |
| TEK | Q02763 | Angiopoietin-1 receptor |
| TGFB1 | P01137 | Transforming growth factor beta-1 proprotein |
| THBS2 | P35442 | Thrombospondin-2 |
| THPO | P40225 | Thrombopoietin |
| TIMP1 | P01033 | Metalloproteinase inhibitor 1 |
| TIMP2 | P16035 | Metalloproteinase inhibitor 2 |
| TLR3 | O15455 | Toll-like receptor 3 |
| TNF | P01375 | Tumor necrosis factor |
| TNFRSF11A | Q9Y6Q6 | TNF receptor superfamily member 11a |
| TNFRSF11B | O00300 | Tumor necrosis factor receptor superfamily member 11B |
| TNFRSF13B | O14836 | Tumor necrosis factor receptor superfamily member 13B |
| TNFRSF13C | Q96RJ3 | Tumor necrosis factor receptor superfamily member 13C |
| TNFRSF14 | Q92956 | Tumor necrosis factor receptor superfamily member 14 |
| TNFRSF17 | Q02223 | Tumor necrosis factor receptor superfamily member 17 |
| TNFRSF18 | Q9Y5U5 | Tumor necrosis factor receptor superfamily member 18 |
| TNFRSF1A | P19438 | Tumor necrosis factor receptor superfamily member 1A |
| TNFRSF1B | P20333 | Tumor necrosis factor receptor superfamily member 1B |
| TNFRSF21 | O75509 | Tumor necrosis factor receptor superfamily member 21 |
| TNFRSF4 | P43489 | Tumor necrosis factor receptor superfamily member 4 |
| TNFRSF8 | P28908 | Tumor necrosis factor receptor superfamily member 8 |
| TNFRSF9 | Q07011 | Tumor necrosis factor receptor superfamily member 9 |
| TNFSF10 | P50591 | Tumor necrosis factor ligand superfamily member 10 |
| TNFSF11 | O14788 | Tumor necrosis factor ligand superfamily member 11 |
| TNFSF12 | O43508 | Tumor necrosis factor ligand superfamily member 12 |
| TNFSF13 | O75888 | TNF superfamily member 13 |
| TNFSF14 | O43557 | Tumor necrosis factor ligand superfamily member 14 |
| TNFSF15 | O95150 | Tumor necrosis factor ligand superfamily member 15 |
| TNFSF18 | Q9UNG2 | Tumor necrosis factor ligand superfamily member 18 |
| TNFSF4 | P23510 | Tumor necrosis factor ligand superfamily member 4 |
| TNFSF8 | P32971 | Tumor necrosis factor ligand superfamily member 8 |
| TNFSF9 | P41273 | Tumor necrosis factor ligand superfamily member 9 |
| TREM1 | Q9NP99 | Triggering receptor expressed on myeloid cells 1 |
| TREM2 | Q9NZC2 | Triggering receptor expressed on myeloid cells 2 |
| VCAM1 | P19320 | Vascular cell adhesion protein 1 |
| VEGFA | P15692 | Vascular endothelial growth factor A |
| VEGFC | P49767 | Vascular endothelial growth factor C |
| VEGFD | O43915 | Vascular endothelial growth factor D |
| VSNL1 | P62760 | Visinin-like protein 1 |
| VSTM1 | Q6UX27 | V-set and transmembrane domain containing 1 |
| WNT16 | Q9UBV4 | Wnt family member 16 |
| WNT7A | O00755 | Protein Wnt-7a |
| PGF | P49763 | Placenta growth factor |
| TGFB3 | P10600 | Transforming growth factor beta-3 proprotein |
| TSLP | Q969D9 | Thymic stromal lymphopoietin |

Targets separated by a line (|) means that the antibody pair used detects the complex containing both subunits, whereas targets separated by a semicolon (;) means co-detection without distinguishing between them.

## **Table S3** Overlapping targets between NULISA ‘CNS Disease Panel’ 120 and ‘Inflammation Panel’ 250.

| **Target** | **Protein ID** | **Protein name** |
| --- | --- | --- |
| CCL11 | P51671 | Eotaxin |
| CCL13 | Q99616 | C-C motif chemokine 13 |
| CCL17 | Q92583 | C-C motif chemokine 17 |
| CCL2 | P13500 | C-C motif chemokine 2 |
| CCL22 | O00626 | C-C motif chemokine 22 |
| CCL26 | Q9Y258 | C-C motif chemokine 26 |
| CCL3 | P10147 | C-C motif chemokine 3 |
| CCL4 | P13236 | C-C motif chemokine ligand 4 |
| CD40LG | P29965 | CD40 ligand |
| CHI3L1 | P36222 | Chitinase-3-like protein 1 |
| CRP | P02741 | C-reactive protein |
| CSF2 | P04141 | Granulocyte-macrophage colony-stimulating factor |
| CX3CL1 | P78423 | Fractalkine |
| CXCL1 | P09341 | Growth-regulated alpha protein |
| CXCL10 | P02778 | C-X-C motif chemokine 10 |
| CXCL8 | P10145 | Interleukin-8, IL8 |
| FGF2 | P09038 | Fibroblast growth factor 2 |
| FLT1 | P17948 | Vascular endothelial growth factor receptor 1 |
| GDF15 | Q99988 | Growth/differentiation factor 15 |
| GFAP | P14136 | Glial fibrillary acidic protein |
| ICAM1 | P05362 | Intercellular adhesion molecule 1 |
| IFNG | P01579 | Interferon gamma |
| IL10 | P22301 | Interleukin-10 |
| IL12p70 | P29459\|P29460 | Interleukin-12 subunit beta\|Interleukin-12 subunit alpha |
| IL13 | P35225 | Interleukin-13 |
| IL15 | P40933 | Interleukin-15 |
| IL16 | Q14005 | Pro-interleukin-16 |
| IL17A | Q16552 | Interleukin-17A |
| IL18 | Q14116 | Interleukin-18 |
| IL1B | P01584 | Interleukin-1 beta |
| IL2 | P60568 | Interleukin-2 |
| IL33 | O95760 | Interleukin 33 |
| IL4 | P05112 | Interleukin-4 |
| IL5 | P05113 | Interleukin-5 |
| IL6 | P05231 | Interleukin-6 |
| IL6R | P08887 | Interleukin-6 receptor subunit alpha |
| IL7 | P13232 | Interleukin-7 |
| IL9 | P15248 | Interleukin-9 |
| KDR | P35968 | Vascular endothelial growth factor receptor 2 |
| NGF | P01138 | Beta-nerve growth factor |
| PGF | P49763 | Placenta growth factor |
| S100A12 | P80511 | Protein S100-A12 |
| TAFA5 | Q7Z5A7 | Chemokine-like protein TAFA-5 |
| TEK | Q02763 | Angiopoietin-1 receptor |
| TNF | P01375 | Tumor necrosis factor |
| TREM1 | Q9NP99 | Triggering receptor expressed on myeloid cells 1 |
| TREM2 | Q9NZC2 | Triggering receptor expressed on myeloid cells 2 |
| VCAM1 | P19320 | Vascular cell adhesion protein 1 |
| VEGFA | P15692 | Vascular endothelial growth factor A |
| VEGFD | O43915 | Vascular endothelial growth factor D |
| VSNL1 | P62760 | Visinin-like protein 1 |

Targets separated by a line (|) means that the antibody pair used detects the complex containing both subunits.

## **Fig S1** Correlation Simoa versus NULISA results.


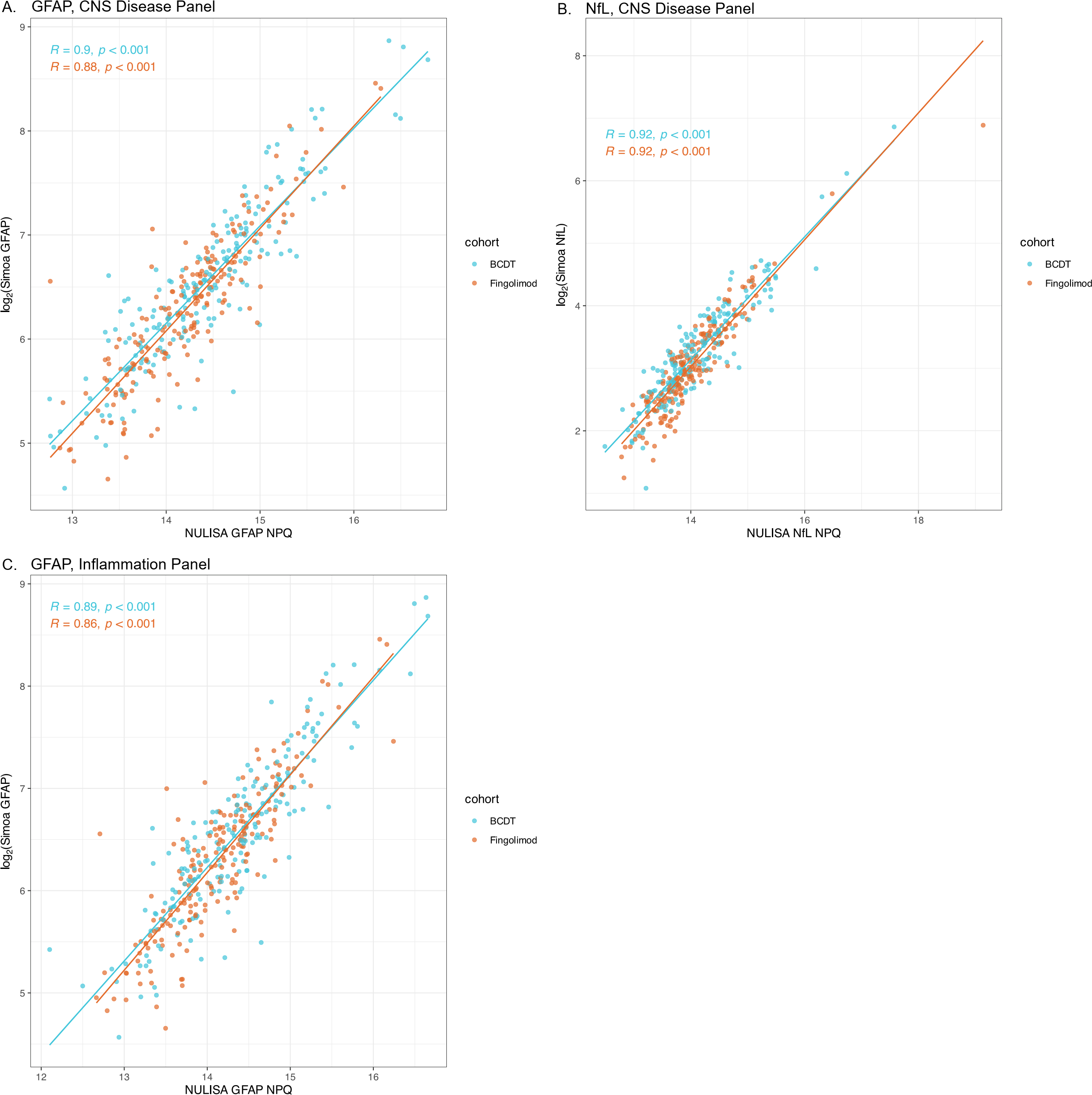


Legend: Person correlation between (A.) log2-transformed Simoa GFAP (serum) and GFAP NPQ (NULISA ‘CNS Disease Panel’), (B.) log2-transformed Simoa GFAP (serum) and GFAP NPQ (NULISA ‘Inflammation Panel’), and (C.) log2-transformed Simoa NfL (serum) and NfL NPQ (NULISA ‘CNS Disease Panel’) in BCDT and fingolimod cohort.

Abbreviations: BCDT, B cell-depleting therapy; GFAP, glial fibrillary acidic protein; NfL, neurofilament light chain; NULISA, NUcleic acid Linked Immuno-Sandwich Assay; Simoa, Single molecule array.

## **Supplementary References**

1. The Swiss MS Cohort. RC2NB. The Swiss MS Cohort, <https://smsc.ch/> (2012, accessed 2024-10-20 2024).

2. Disanto G, Benkert P, Lorscheider J, et al. The Swiss Multiple Sclerosis Cohort-Study (SMSC): A Prospective Swiss Wide Investigation of Key Phases in Disease Evolution and New Treatment Options. *PloS one* 2016; 11: e0152347. 20160331. DOI: 10.1371/journal.pone.0152347.

3. Teunissen CE, Petzold A, Bennett JL, et al. A consensus protocol for the standardization of cerebrospinal fluid collection and biobanking. *Neurology* 2009; 73: 1914-1922. 2009/12/02. DOI: 10.1212/WNL.0b013e3181c47cc2.

4. Kurtzke JF. Rating neurologic impairment in multiple sclerosis: an expanded disability status scale (EDSS). *Neurology* 1983; 33: 1444-1452. 1983/11/01.

5. Neurostatus-UHB. Ltd c/o University Hospital Basel Switzerland, <https://www.neurostatus.net/> (2016, accessed 2024-11-02 2024).

6. McDonald WI, Compston A, Edan G, et al. Recommended diagnostic criteria for multiple sclerosis: guidelines from the International Panel on the diagnosis of multiple sclerosis. *Annals of neurology* 2001; 50: 121-127. 2001/07/18.

7. Kappos L, Butzkueven H, Wiendl H, et al. Greater sensitivity to multiple sclerosis disability worsening and progression events using a roving versus a fixed reference value in a prospective cohort study. *Multiple sclerosis (Houndmills, Basingstoke, England)* 2018; 24: 963-973. 20170530. DOI: 10.1177/1352458517709619.

8. Feng W, Beer JC, Hao Q, et al. NULISA: a proteomic liquid biopsy platform with attomolar sensitivity and high multiplexing. *Nat Commun* 2023; 14: 7238. 20231109. DOI: 10.1038/s41467-023-42834-x.
